# The Gapes

**Published:** 1870-04

**Authors:** 


### THE GAPES.

This is a physical disease as to chickens, and moral as to man; a kind of social crime, for it shows a contemptuous indifference to "present company;" it is a sign of sleepiness, or weariness, or stupidity, for a man may gape under the most powerful preaching, or in hearing a philosophical lecture on the most absorbing subject, but too "deep" for his mental fathoming.

There is always a certain cure for the gapes in chickens, because it is a malady caused by the presence of one or more little red worms in the top of the windpipe of the little chick; they can be easily fished out one by one, by opening the mouth, squeezing the windpipe a little between the thumb and finger, then looking down, these flesh-colored worms will be found clinging to the side of the windpipe; take a horse-hair.

make a little loop, thrust it down, twist it around among the worms, and when entangled draw it up, and thus continue until none are left; the cure is immediate, perfect, and permanent. If neglected, the worms increase rapidly and choke the chicken to death.

In man the gapes is a disease of the head, a want of brains and is incurable.

